# Prevalence and barriers to HIV testing among mothers at a tertiary care hospital in Phnom Penh, Cambodia. Barriers to HIV testing in Phnom Penh, Cambodia

**DOI:** 10.1186/1471-2458-10-494

**Published:** 2010-08-18

**Authors:** Yuri Sasaki, Moazzam Ali, Vong Sathiarany, Koum Kanal, Kazuhiro Kakimoto

**Affiliations:** 1 School of International Health, Graduate School of Medicine, The University of Tokyo, Japan; 2 World Health Organization, Geneva, Switzerland; 3 The National Maternal and Child Health Center, Phnom Penh, Cambodia; 4 School of Nursing & Graduate School of Nursing, Osaka Prefecture University, Japan

## Abstract

**Background:**

One-third of all new HIV infections in Cambodia are estimated to be due to mother-to-child transmission. Although the Ministry of Health adopted a policy of provider-initiated HIV testing and counseling (PITC), nearly a quarter of pregnant mothers were not tested in 2007. Greater acceptance of HIV testing is a challenge despite Cambodia's adoption of the PITC policy.

**Methods:**

A hospital-based quantitative and cross-sectional survey was conducted to assess the prevalence of and barriers to HIV testing among mothers after delivery at the National Maternal and Child Health Center in Phnom Penh. The Center is one of the largest maternal and child care hospitals in the country to offer PITC services. All 600 eligible mothers who were admitted to the hospital after delivery from October to December 2007 were approached and recruited. Data were collected via a semi-structured questionnaire.

**Results:**

The prevalence of HIV testing among women who delivered at the hospital was 76%. In multivariate logistic regression, factors such as the perceived need to obtain a partner's permission to be tested (OR=0.27, 95% CI=0.14-0.51, p<0.01), the lack of knowledge about HIV prevention and treatment (OR=0.38, CI=0.22-0.66, p<0.01), and the lack of access to ANC services (OR=0.35, 95% CI=0.21-0.58, p<0.01) were found to be the main barriers to HIV testing.

**Conclusion:**

To achieve greater acceptance of HIV testing, counseling on HIV prevention and treatment must be provided not only to mothers but also to their partners. In addition, utilization of non-laboratory staff such as midwives to provide HIV testing services in rural health facilities could lead to the greater acceptance of HIV testing.

## Background

In Cambodia, HIV prevalence among people ages 15-49 declined from 3.2% in 1997 to 0.6% in 2005 [1]. The government's policy of supporting 100% condom use in commercial sex establishments and in providing sex workers with access to affordable condoms is widely credited with the reversal of this trend [2]. However, the incidence of HIV infection among women attending antenatal care (ANC) has not declined [3]. Therefore, the major mode of transmission has shifted to mother-to-child transmission, and one-third of all new HIV infections in Cambodia are estimated to be due to mother-to-child transmission [4].

In response, the Ministry of Health (MoH) developed a national policy on the prevention of mother-to-child transmission (PMTCT) of HIV in 2001; this policy highlights the importance of counseling as well as HIV testing in order to inform not only HIV-positive mothers but also HIV-negative mothers about HIV transmission. As PMTCT services have involved all levels of the community since the program was piloted at the National Maternal and Child Health Center (NMCHC) in Phnom Penh, the capital of Cambodia [5], the acceptance of HIV testing among pregnant women in ANC has increased. Of the first attendees at ANC with PMTCT services, 53.1% were tested for HIV in 2005 [6], 69.3% were tested in 2006 [7], and 76.7% were tested in 2007 [8]. However, nearly a quarter of pregnant mothers were not tested in 2007 although the MoH adopted a policy of provider-initiated HIV testing and counseling (PITC) [9] in 2006.

HIV testing and counseling are crucial to the prevention of new HIV infections, including mother-to-child transmission, and to the early initiation of treatment and care. However, the greater acceptance of HIV testing and counseling is still a challenge for Cambodia. Thus, the objective of this study was to investigate the prevalence of and barriers to HIV testing among mothers as part of a PITC strategy at a tertiary care hospital in Phnom Penh. The outcome variable was greater acceptance of HIV testing among these mothers.

## Methods

A hospital-based quantitative and cross-sectional survey to assess the prevalence of and barriers to HIV testing among mothers after delivery was conducted at NMCHC in Phnom Penh, which is one of the largest maternal and child care hospitals offering PITC services as well as ANC services.

Deliveries per month are almost constant year-round at NMCHC, and this study was conducted from October to December 2007. Because of the adoption of the PITC policy at the NMCHC, mothers who delivered there were offered HIV testing. Most mothers who had a normal vaginal delivery or who delivered via a caesarean section normally stayed at the hospital for two days or seven days, respectively. All mothers who delivered at NMCHC and who were admitted there during the study period were approached and recruited.

The participants were asked to participate in a half-hour face-to-face interview with trained interviewers. Questionnaire items were pre-tested in focus groups of ten women in the maternity ward before this study was conducted. The questionnaire was tested for its wording and its feasibility during the pilot test. It was first developed in English to receive comments from experts. After it was translated into the Khmer language, it was then back-translated into English to ascertain the clarity and consistency of the Khmer version (Additional file 1).

The questionnaire covered information on the socio-demographic characteristics of the mother and her partner, the mother's experience with ANC and HIV testing, the mother's sources of information on HIV testing, the mother's knowledge about HIV prevention and treatment, and the perceived need to obtain her partner's permission to be tested for HIV. The subject who made antenatal visit to public or private hospitals, clinic or health center for pregnancy health check, fetal development and risk assessment in pregnancy was defined as an ANC experienced one.

Maternal knowledge about HIV prevention and treatment was examined by asking nine questions (Table 1). Since the questions on HIV prevention and treatment were very basic, mothers were evaluated as knowledgeable if they achieved a 'perfect score,' i.e. they correctly answered all questions.

**Table 1:** Questions to measure maternal knowledge about HIV prevention and treatment

A person can be at risk of HIV infection if she/he does not use a condom consistently.
A healthy-looking person can be infected with HIV.
A person can be infected with HIV by a mosquito bite.
A person can be infected with HIV by sharing meals with someone infected with HIV.
An HIV-infected mother can transmit HIV to her baby during pregnancy.
An HIV-infected mother can transmit HIV to her baby during delivery.
An HIV-infected mother can transmit HIV to her baby through breast milk.
All babies will be HIV-positive if their mothers are HIV-positive.
There are special medications that can be given to a woman infected with HIV to reduce the risk of transmission to her baby.

The ethics review committee of the MoH in Cambodia approved the research protocol and the survey instrument. A one-day training course on research protocols, administration of the questionnaire, and ethics was provided for field research assistants. A small pilot study was carried out before the main survey.

Written informed consent was obtained from respondents at the beginning of the interview after the research was explained. In light of the sensitivity of the topic, special precautions were taken to ensure respondent confidentiality. HIV testing and counseling were offered to those mothers who had not undergone HIV testing. In order to avoid response bias in the study, however, the reasons for not being tested were not determined: respondents may not make a response by true reasons, but by self-defensive reasons.

All data were entered into SPSS 11.0 statistical software for analysis. Frequencies and basic descriptive statistics were calculated for all variables, including cross tabs to determine potential relationships between key variables. A Mann-Whitney U-test was used to examine continuous variables, and a Chi-square test and Fisher's exact test were used to examine categorical data. A p*-*value of less than 0.05 was considered statistically significant. For multivariate logistic regression analysis, the dependent variable was whether the respondent had taken a HIV test at the hospital. Independent variables included maternal and paternal educational and occupational backgrounds, frequency of visits and access to ANC, the perceived need to obtain a partner's permission to be tested, and knowledge about HIV prevention and treatment.

## Results

A total of 600 women underwent delivery and were admitted to the hospital during the study period. One form was rejected as information was incomplete, so information from 599 clients was included in the final analysis.

Of the 599 mothers in the study, 455 (76%) were tested for HIV (Table 2). The major sources of information on HIV testing were health care providers (360: 79.1%) and mass media (86: 18.9%).

**Table 2:** Comparison of mothers who have been tested and those who have not been tested in terms of socio-economic characteristics

	Total n = 599(100%)	Previously tested for HIV n = 455(76%)	Never tested n = 144(24%)	p-value
Mean age (SD)	27.1(5.8)	27.1(5.4)	27.0(6.8)	0.297^a^
Marital status				
Married	588(98.7)	448(98.7)	139(98.6)	0.996^b^
Divorced	4(0.7)	3(0.7)	1(0.7)	
Other	4(0.7)	3(0.7)	1(0.7)	
Primiparous				
Yes	305(53.9)	230(53.2)	75(56)	0.58 ^b^
No	261(46.1)	202(46.8)	59(44)	
Level of education				
≤Y6	330(55.2)	238(52.3)	92(64.3)	0.012^b^
> Y6	268(44.8)	217(47.7)	51(35.7)	
Occupation				
Company employee	275(46.3)	225(49.8)	50(35.2)	< 0.001^b^
Otherwise employed	89(15)	46(10.2)	43(30.3)	
Other	230(38.7)	181(40)	49(34.5)	
Partner's level of education				
≤Y6	186(31.3)	118(26.2)	68(47.2)	< 0.001^b^
> Y6	409(68.7)	333(73.8)	76(52.8)	
Partner's occupation				
Company employee	335(56.8)	270(60.3)	65(45.8)	< 0.001^b^
Otherwise employed	160(27.1)	100(22.3)	60(42.3)	
Other	95(16.1)	78(17.4)	17(12)	
Information source on HIV testing (multiple)				
Health care provider		360(79.1)		
Family member		20(4.4)		
Friend		7(1.5)		
Mass media		86(18.9)		
Other		19(4.2)		

SD, standard deviation

a: Mann-Whitney test

b: Chi-square test

### Socio-demographic characteristics of the tested mothers and non-tested mothers

The 455 mothers who were tested for HIV had a mean age (SD) of 26 years (±5.4) while the 144 mothers who were not tested had a mean age of 25 years (±6.8). Of those tested, 230 (53.2%) were primiparas; of those not tested, 75 (56%) were primiparas (p = 0.58) (Table 2). Both the mother's educational level (p < 0.05) and occupation (p < 0.001) were significantly associated with having been HIV tested. The partner's educational level (p < 0.001) and occupation (p < 0.001) were also significantly associated with the mother having been HIV tested.

### ANC visits, HIV knowledge, and the perceived need to obtain a partner's permission to be tested for mothers who were tested for HIV and those who were not

Among the mothers who were tested for HIV, 399 (87.9%) visited ANC more than two times; among those who were not tested, 94 (66.2%) did so (p < 0.001) (Table 3). Two hundred and eighty-seven mothers (63.8%) who were tested visited ANC in Phnom Penh at least once. In contrast, 34 (27.2%) of those who were not tested visited ANC in Phnom Penh at least once (p < 0.001).

**Table 3:** Comparison of mothers who have been tested and those who have not been tested in terms of ANC, HIV knowledge, and partners' perspective on HIV testing

	Total n = 599(100%)	HIV tested n = 455(76%)	Never tested n = 144(24%)	p-value
Frequency of ANC visits during this pregnancy				
None	19(3.2)	4(0.9)	15(10.6)	< 0.001
Once	23(3.9)	13(2.9)	10(7.0)	
Twice	61(10.2)	38(8.4)	23(16.2)	
> 2 times	493(82.7)	399(87.9)	94(66.2)	
ANC with				
Myself	136(24.1)	103(23.5)	33(26.2)	0.258
Husband	387(68.5)	307(69.9)	80(63.5)	
Someone else	42(7.4)	29(6.6)	13(10.3)	
Location of ANC				
In Phnom Penh	321(55.8)	287(63.8)	34(27.2)	< 0.001
Outside Phnom Penh	254(44.2)	163(36.2)	91(72.8)	
Perfect score**^§ ^**(knowledge)				
Yes	232(40.9)	206(46.5)	26 (21)	< 0.001
No	335(59.1)	237(53.5)	98(79)	
Perceived need to obtain a partner's permission to be tested				
No need	454(76.3)	421(92.7)	103(73)	< 0.001
Permission needed	141(23.7)	33(7.3)	38(27)	

p value for Chi-square test

**^§^**Correctly answered all questions

Among mothers who were tested for HIV, 206 (46.5%) correctly answered the nine questions (Table 1) which measured basic knowledge about HIV prevention and treatment based on our definition (p5), and it was compared to 26 (21%) among mothers who were not (p < 0.001) (Table 3). Very few of the mothers who were tested, i.e. 33 (7.3%), perceived the need to obtain their partner's permission to be tested in contrast to the sizable number of mothers who were not tested, i.e. 38 (27%), but who perceived the need to obtain that permission (p < 0.001).

### Barriers to HIV testing

After adjustments, multivariate logistic regression analysis revealed that the potential barriers to HIV testing were the perceived need to obtain a partner's permission to be tested (OR = 0.27, 95% CI = 0.14-0.51, p < 0.01), lack of knowledge about HIV prevention and treatment (OR = 0.38, CI = 0.22-0.66, p < 0.01), and lack of access to ANC services (OR = 0.35, 95% CI = 0.21-0.58, p < 0.01) (Table 4).

**Table 4:** Barriers to undergoing HIV testing according to logistic regression (n = 524)

Variables		Adjusted
	OR	95%CI
**Level of education**		
≤Y6	1.00	
> Y6	0.78	0.47-1.30
**Occupation**		
Company employee	1.00	
Otherwise employed	0.64	0.31-1.33
Other	0.79	0.46-1.36
**Partner's level of education**		
≤Y6	1.00	
> Y6	1.27	0.74-2.17
**Partner's occupation**		
Company employee	1.00	
Otherwise employed	0.66	0.37-1.17
Other	1.07	0.51-2.23
**Frequency of ANC visits during this pregnancy**		
Once	1.00	
Twice	0.45	0.12-1.78
> 2 times	1.08	0.31-3.73
**Location of ANC (access)**		
In Phnom Penh	1.00	
Outside Phnom Penh	0.35	0.21-0.58**
**Perceived need to obtain a partner's permission to be tested**		
No need	1.00	
Permission needed	0.27	0.14-0.51**
**Perfect score^§ ^(knowledge)**		
Yes	1.00	
No	0.38	0.22-0.66**

CI, confidence interval; OR, odds ratio.

*p < 0.05; **p < 0.01

**^§^**Correctly answered all questions

## Discussion

This is the first epidemiological study in Cambodia to assess the prevalence of and barriers to HIV testing after implementation of the PITC initiative. Most such studies have been done in African countries. Out of the mothers in this study, 76% were tested for HIV. This indicates that almost a quarter of the targeted mothers declined testing as part of the PITC strategy.

In some African countries, the acceptance of HIV testing among pregnant women after the adoption of a PITC strategy exceeded 90% [10-12]. For example, a study in urban clinics in Zimbabwe [11]found that 99.9% of women present for ANC were tested for HIV as part of a PITC strategy, compared to 65% who were tested during voluntary counseling and testing (VCT). That said, the PITC strategy must be evaluated by comparing the incidence of adverse effects such as domestic violence experienced by mothers who opted-in to HIV testing [13]. A study in Botswana [12]found that the acceptance of HIV testing increased from 75.3% to 90.5% after adoption of a PITC strategy. In comparison, mothers in Cambodia face greater barriers to the acceptance of HIV testing that exceed the effects of the PITC strategy.

One barrier was the perceived need to obtain a partner's permission to be tested. Maman [14]found that the fear of a partner's reaction to an HIV-positive result was an important barrier to HIV testing in Tanzania, and Dahl [15]also found that the one of the most common reasons for test refusal among pregnant women was the need to discuss the issue with a partner beforehand in Uganda. However, a study conducted prior to the introduction of PITC at the same facility indicated that a partner's permission was not a significant barrier to HIV testing when HIV testing was offered in a VCT approach where male involvement was strongly encouraged in the form of group education and individual counseling sessions [16]. Although simply comparing these results to the current results is not necessarily appropriate, the current results may be due to the simplification of test counseling. There is no doubt that PITC is a very effective strategy to foster greater acceptance of HIV testing, but involvement of male partners in PMTCT services such as group education and individual counseling still seems to be an important way to make partners aware of the benefits of HIV testing and help them better understand HIV testing by mothers even with a PITC approach. Although no effective interventions to improve partner attendance during ANC have been noted, women attending ANC in Kenya were asked to return with their male partner for individual or couple VCT [17]. Sixteen-percent of women returned to ANC with their partners, and over 95% of the men attending ANC accepted HIV testing. Partner attendance during ANC was still a challenge, but the results implied that offering HIV testing and counseling services to men with options for couple and individual counseling during ANC are an acceptable strategy to increasing male involvement in PMTCT and promote male HIV testing.

Maternal knowledge about HIV prevention and treatment is a well-known factor related to the acceptance of HIV testing [18,19]. The current results that ascertained basic knowledge about HIV prevention and treatment agreed with results from other studies. Communication strategies as well as counseling as part of PMTCT services could provide opportunities for mothers to learn about HIV prevention and treatment. However, a point worth mentioning is that only 46.5% of those mothers who were tested for HIV scored perfectly although the questions mothers were asked in this study were very basic. This suggests that many mothers receive HIV testing despite a lack of understanding about the advantages and disadvantages of HIV testing. This may lead to missing opportunities to receive adequate information and may even lead to refusal to undergo HIV testing. In Botswana, 68% of individuals who were tested for HIV under a national policy of PITC believed that they were not able to refuse the test when it was offered [20]. In rural Zimbabwe, 55% of women who accepted HIV testing directly after group education as part of their routine ANC blood tests were not aware of the possibility of opting for individual pre-test counseling [21].

The guidelines on PITC state that greater knowledge of one's HIV status is critical to increasing access to HIV treatment, care, and support in a timely manner, and such knowledge offers people living with HIV the opportunity to receive information and tools to prevent HIV transmission to others [22]. However, WHO/UNAIDS have reduced the emphasis on counseling in their revised testing guidelines, which include simplified pre-test counseling. For example, individual risk assessment and risk reduction plans are not covered during pre-test counseling, and pre-test information were only provided in the form of group health information talk rather than individual counseling session after adopting PITC strategy in Cambodia. Therefore, our result that more than half of mothers could not answer basic questions correctly about HIV prevention and treatment is considered to be caused by the simplified counseling as part of a PITC strategy. In addition, they may not know that they have the right to have additional information if they are susceptible to coercion to be tested. Introducing a PITC strategy may increase HIV testing, but mothers must understand basic information about HIV, including their right not to be tested.

Mothers who received ANC outside Phnom Penh had less chance of undergoing HIV testing than did mothers who received ANC in Phnom Penh. The number of testing sites around the country have increased and the number of people tested at licensed sites, which have rapid test kits and offer free counseling and HIV testing, increased from 1,766 in 1997 to 152,147 in 2005 [23]. Nevertheless, HIV testing and counseling services are not as available outside Phnom Penh as they are within the city. The 2005 Cambodian Demographic and Health Survey [1]found that less than 70% of pregnant women received ANC from trained personnel in rural areas, as compared to 80% who received it in urban areas. Numbers of ANC visits also differed. Less than a quarter of the mothers living in rural areas received ANC more than four times while nearly half of the mothers living in urban areas did [1]. Mothers who received ANC outside Phnom Penh may have fewer opportunities to learn about HIV testing than do mothers who received ANC in Phnom Penh. As there are few data on the difference in ANC services in urban and rural areas of Cambodia, further studies on the quality of ANC in both urban and rural areas are needed.

However, a previous study showed that one of the differences was HIV services [24]. Because the Cambodian regulations allow only laboratory technicians to perform HIV testing and these technicians are not assigned to all health facilities in rural areas, mothers who receive ANC in rural areas must lack the opportunity to be tested for HIV. Kanal [24]showed that half-day training for non-laboratory staff such as midwives was a feasible way to provide sufficient proficiency to implement HIV testing. HIV testing should be made available in all health facilities in rural areas through strategic approaches such as efficient utilization of human resources.

Although this study provided important and useful information on the prevalence of HIV testing and it highlighted barriers that might hamper the acceptance of HIV testing services, it has some limitations. The study is hospital-based and does not include those mothers who delivered at other locations such as private facilities or at home. In rural areas, more than 80% of mothers deliver at home, and in Phnom Penh 40% of mothers deliver at private facilities (21.6%) or at home (21.6%) [1]. Results of the current study may not be generalized and may only be applicable to the study site or to other major hospitals offering PITC services. That said, approximately 40% of babies in Phnom Penh are born at the current study site [25]. In the future, a community-based investigation must be performed with a focus on barriers to use of services by mothers who give birth either at home or at other institutions., Perez suggests that in rural Zimbabwe knowledge about PMTCT must be enhanced among traditional birth attendants and that these individuals must be integrated into the health system in order to improve access to PMTCT services for mothers delivering at home [26]. Interventions such as birth attendant training and home visits by trained health professionals must be considered in Cambodia.

## Conclusions

This study was designed to investigate the prevalence of and barriers to HIV testing as part of a PITC strategy. Focusing on mothers' characteristics and their history of ANC, this study demonstrated that the perceived need to obtain a partner's permission, lack of knowledge about HIV prevention and treatment, and lack of access to ANC services were barriers to HIV testing. Education in HIV prevention, treatment and counseling must be provided not only to mothers but also to their partners even when a PITC approach is adopted. In addition, utilization of non-laboratory staff such as midwives to provide HIV testing services in rural health facilities could lead to the greater acceptance of HIV testing.

## Competing interests

The authors declare that they have no competing interests

## Authors' contributions

YS, MA, and KKak carried out data analysis and drafted this manuscript. KKan and VS helped to collect data and participated in coordinating the study design to involve trained interviewers. YS and KKak helped with the design of this study. All authors read and approved the final manuscript.

## Pre-publication history

The pre-publication history for this paper can be accessed here:

http://www.biomedcentral.com/1471-2458/10/494/prepub

## Supplementary Material

Additional file 1 **Questionnaire**. Participants in this study were asked to participate in a half-hour face-to-face interview using the Khmer version of this questionnaire. Click here for file
