# Supplementary material for: Prevalence and barriers to HIV testing among mothers at a tertiary care hospital in Phnom Penh, Cambodia. Barriers to HIV testing in Phnom Penh, Cambodia
Source: BMC Public Health. 2010 Aug 18;10:494. doi: 10.1186/1471-2458-10-494 (PMC2930599; doi:10.1186/1471-2458-10-494)
Supplement: Additional file 1 — Questionnaire. Participants in this study were asked to participate in a half-hour face-to-face interview using the Khmer version of this questionnaire. [file 1471-2458-10-494-S1.DOC]

Name of interviewer:………...........………………

Date of interview:……………………………..........

Code number:.............................................

**Questionnaire**

**BASIC INFORMATION**

| 1. Age |  |
| --- | --- |
| 1. Address | □ Daun Penh District  □ Tuol Kork District  □ Chamkaman District  □ Russeykeo District  □ 7 Makara District  □ Meanchey District  □ Dangkor District  □ Provinces................................................ |
| 1. Marital Status | □ Married □ Widowed □ Others................. |
| 1. Religion | □ Buddhism  □ Muslim  □ Christian  □ Other:_______________________ |
| 1. Number of Children | Living children(including this baby)..................  Abortion (including miscarrage)......................  Dead children................................................. |
| 1. Education | □ No education  □ Primary (1-6yrs)  □ Lower secondary (7-9yrs)  □ Upper secondary (10-12 yrs)  □ Higher (University/post university) |
| 1. Occupation | □ Farmer  □ Worker  □ Merchant  □ Fisherman  □ Artist  □ Government official  □ Motortaxis/taxi driver  □ Others……………. |
| 1. Average individual monthly income (USD) |  |
| 1. Average family monthly income (USD) |  |
| 1. Living arrangement | □ Husband □ Parents □ Other:___________ |
| 1. How many family members do you have? | ________________________ |

**PREGNANCY-RELATED QUESTIONS**

| 1. Was this pregnancy planned? | □ Yes □ No |
| --- | --- |
| 1. Do you plan to have more babies?   **If yes**, how many children you need?  **If no**, what family planning method you want to use? | □ Yes □ No  ________________  ________________ |
| 1. Have you ever visited public or private hospitals, clinic or health center for ANC, and seen by doctors, midwives or nurses for this pregnancy?   **If yes**, how often?    Who accompanied you to the clinic?  What clinic did you visited? | □ Yes □ No  □ Once □ Twice □ >Twice  □ Alone □ Husband □ Others........  □ NMCHC  □ Calmette  □ Private clinic  □ HC within Phnom Penh  □ HC outside Phnom Penh  □ Others............................. |
| 1. Have you ever visited ANC before?   (For 1^st^ pregnancy, there is no need to ask this question) | □ Never  □ Once  □ Twice  □ >Twice |

**HIV KNOWLEDGE &ATTITUDE**

**Knowledge on HIV**

| 1. A person can be at risk of HIV infection if she/he does not use a condom consistently. | □ Yes □ No |
| --- | --- |
| 1. A healthy-looking person can be infected with HIV. | □ Yes □ No |
| 1. A person can be infected with HIV by a mosquito bite. | □ Yes □ No |
| 1. A person can be infected with HIV by sharing meals with someone infected with HIV. | □ Yes □ No |
| 1. An HIV-infected mother can transmit HIV to her baby during pregnancy. | □ Yes □ No |
| 1. An HIV-infected mother can transmit HIV to her baby during delivery. | □ Yes □ No |
| 1. An HIV-infected mother can transmit HIV to her baby through breast milk. | □ Yes □ No |
| 1. All babies will be HIV-positive if their mothers are HIV-positive. | □ Yes □ No |
| 1. There are special medications that can be given to a woman infected with HIV to reduce the risk of transmission to her baby. | □ Yes □ No |

**Attitudes toward HIV testing**

| 26. Have you ever tested for HIV?  27. Did you have HIV test during this pregnancy?    28. How did you know about HIV testing?  29. Where did you do HIV test?          30. Was HIV test volunteer or compulsory?  31. Did you get the test result? | □ Yes □ No (skip to 32)  □ Yes □ No  □ Physician  □ ANC Counselor  □ Family  □ Neighbor  □ Friend  □Private Clinic  □ Mass media  □ Other:___________________  □ Public hospital  □ Private clinic  □ VCCT  □ Health Centre..........................  □ Others.....................................  □ Volunteer  □ Compulsory  □ Other...............................  □ Yes □ No |
| --- | --- |
| 32. Can you do HIV testing without permission from your husband? (ask all women) | □ Yes □ No |
| 33. Can you ask your husband to use condom? | □ Yes □ No |

**HUSBAND INFORMATION**

| 34. Level of education | □ No education  □ Primary (1-6)  □ Lower Secondary (7-9)  □ Upper Secondary (10-12)  □ Tertiary (>12) |
| --- | --- |
| 35. Occupation | □ Farmer  □ Worker  □ Merchant  □ Fisherman  □ Artist  □ Government official  □ Motortaxis/taxi driver  □ Others……………. |
| 36. Has your husband ever got tested for HIV?  ***If yes*..,**   - When was his last test? | □ Yes □ No □ Don’t know  □ Same day as mine □ Different day from mine  □ Don’t know |
| 37. Do you know your husband test result? | □ Yes □No |

**Baby Information**

| 1. Baby situation | □ Alive  □ Dead  □ Premature |
| --- | --- |
| 1. Delivery method | □ Normal delivery  □ Vacuum extraction  □ C-Section |
| 1. Weight of the baby? | ___________________kg.  ___________________kg. |

THANKS FOR YOUR PARTICIPATION
